# Dietary pattern and risk of hyperuricemia: an updated systematic review and meta-analysis of observational studies

**DOI:** 10.3389/fnut.2024.1218912

**Published:** 2024-02-28

**Authors:** Zhao-Yan Wen, Yi-Fan Wei, Ye-Huan Sun, Wen-Ping Ji

**Affiliations:** ^1^Scientific Research Management Department, Chaohu Hospital Affiliated to Anhui Medical University, Hefei, China; ^2^Department of Clinical Epidemiology, Shengjing Hospital of China Medical University, Shenyang, China; ^3^Department of Epidemiology and Health Statistics, School of Public Health, Anhui Medical University, Hefei, China

**Keywords:** dietary pattern, hyperuricemia, meta-analysis, risk, systematic review

## Abstract

**Objectives:**

The associations of *posteriori* dietary patterns with the risk of hyperuricemia (HUA) are contradictory. Several fair-quality observational studies with inconsistent results have been published following a prior review. Herein, we carried out an updated systematic review and meta-analysis to quantitatively analyze the aforementioned relationships.

**Methods:**

Our analysis protocol has been registered with PROSPEPO (Number: CRD42022376745). English publications were searched in Embase, PubMed, and Web of Science from inception to January 1, 2024. Summary odds ratio (OR) and 95% confidence intervals (CIs) were calculated by a random-effects model. We also conducted subgroup, sensitivity, and meta-regression analyses and publication bias assessments.

**Results:**

Thirteen studies with a total of 163,192 participants were included in the current meta-analysis. Our finding revealed that the plant-based pattern was linked with a 17% decreased risk of HUA (OR = 0.83, 95%CI = 0.72–0.94, *I^2^* = 72.9%, *n* = 10). There was no evidence of publication bias in the present analysis. The results of subgroup analyses were generally consistent with the main findings. In meta-regression analyses, no evidence of heterogeneity was detected in the subgroups. Furthermore, our analyses indicated that the animal-based food pattern (OR = 1.36, 95%CI = 1.25–1.47, *I^2^* = 26.7%, *n* = 11) and sweet food pattern (OR = 1.24, 95%CI = 1.06–1.46, *I^2^* = 0, *n* = 2) was related to an increased risk of HUA.

**Conclusion:**

The plant-based pattern is inversely correlated with HUA risk, whereas animal-based food patterns and sweet food patterns are positively correlated with HUA risk.

## Introduction

Uric acid is an end-product of purine metabolism (1). Excessive production of uric acid and reduced excretion through the kidney are the main causes of hyperuricemia (HUA) (1). Notably, HUA has been reported as the second most frequent metabolic disease after diabetes globally (2). In the United States, a nationwide survey revealed that the prevalence rate of HUA is substantial, with 20.0% of women and 20.2% of men being affected (3). A recent report from China showed that the prevalence rates of HUA increased steadily from 8.5 to 18.4% from 2000 to 2017 (4). Previous studies have suggested that lifestyle (especially diet) and history of cardiovascular disease were related to the incidence of HUA (5–8). Of particular concern is that diet might be the most important and cost-effective factor that can be used to modify the risk of HUA (9).

In nutritional epidemiology, previous studies mainly explored individual nutrient or food intake but did not consider potentially complex interactions among various foods or nutrients (10, 11). In this context, a representative of comprehensive dietary variables, dietary pattern, has emerged to reveal the impact of overall diet (12). Till now, there are two ways to determine dietary patterns, which are the *priori* method and the *posteriori* method. The former is mainly based on dietary guidelines compliance, while the latter is based on dietary data (13). During the last 5 years, a large number of studies have emerged to explore the effect of dietary patterns on the risk of hyperuricemia (14–18); however, the findings have been inconsistent. For example, in the latest cross-sectional study with 4,855 participants, a significantly inverse association between plant-based dietary patterns and HUA risk was observed (15). Conversely, the aforementioned association was non-significant in another cross-sectional study in 2022 including 2,646 subjects (16). In nearly 1 year, several fair-quality studies have been published (19–21), but the results were inconsistent. For example, two studies found that plant-based patterns and animal-food patterns were not associated with the risk of HUA (19, 20).

With the emergence of more new studies with inconsistent results exploring the correlation of dietary patterns with HUA risk, a systematic collection and assessment of the aforementioned results will provide a better understanding of the impact of overall diet on the risk of HUA. Consequently, we carried out this updated systematic review and meta-analysis of available evidence to provide a quantitative evaluation of the association between *posteriori* dietary patterns and HUA risk.

## Methods

### Protocol, registration, reporting standards, and search strategy

We reported this systematic review and meta-analysis following a standardized method with the Preferred Meta-Analysis of Observational Studies in Epidemiology guidelines (Supplementary Table S1) (22) and Reporting Items for Systemic Reviews and Meta-Analyses guidelines (Supplementary Table S2) (23). The protocol for the present review has been registered in the International Prospective Register of Systematic Reviews (registration number: CRD42022376745). All relevant works of literature were searched by two independent individuals (WZY and JWP) in three electronic databases, including Embase, PubMed, and Web of Science from inception up until January 1, 2024. Details of the search strategy are presented in Supplementary Table S3. Furthermore, all references cited in the included literature were manually searched to determine additional publications.

### Study selection

Two authors (WZY and JWP) began by independently and rigorously screening titles and abstracts and then scrutinized full-text articles. Differences were settled by consensus. Articles that met the following criteria were included in the analysis (1): observational study (2); the exposure was evaluated by dietary patterns which were identified using, e.g., factor analysis, cluster analysis, reduced rank regression, and principal component analysis in primary studies (3); the outcome was the risk of hyperuricemia (4); analyses reported risk estimates such as relative risk (RR), prevalence ratio (PR), odds ratio (OR), hazard ratio (HR), and 95% confidence intervals (CIs) or provided relevant data for calculating relative risk and corresponding 95% CI. The exclusion criteria were as follows (1): non-original research like commentaries, editorial review articles, systemic reviews, meta-analyses, animal studies, and meeting abstracts (2); studies that did not report effect value or relevant data for calculation (3); publications that were written in a non-English language.

### Data extraction and quality assessment

Two investigators (WZY and JWP) examined the main features of all eligible studies and extracted the following information: first author’s name, year of publication, study design, total number of subjects, dietary assessment method, dietary patterns identified and food items with high factor loading in each dietary pattern, risk estimates with 95% CIs, covariates matched in the study design, or confounding factors of adjustment in the model. Two tools were applied to evaluate the quality of the included articles. For cross-sectional studies, we applied the Agency for Healthcare Research and Quality (AHRQ) to evaluate study quality (24). This tool consists of 11 domains. Each domain is awarded 1 point for a “Yes” answer and 0 points for “No” or “Unclear” answer. We classified assessment scores of 10–11, 6–9, and < 6 for good, fair, or poor quality of included studies, respectively. For case–control studies and cohort studies, the Newcastle-Ottawa Quality Assessment Scale (NOS) was applied to assess study quality (25). Briefly, this scale was assigned a total of nine stars with three domains, including four stars for selection, two stars for comparability, and three stars for outcomes. Studies that received <4 points represent poor, 4–7 represent fair, and 8–10 represent good; discrepancies in the extracted information and quality assessment were solved by discussion.

### Statistical analysis

In our meta-analysis, RR and HR were considered approximations of OR (26–28). RR was substituted for PR and the prevalence of PR was used to convert RR to OR in the original studies (26). The random-effects model was applied for this meta-analysis because the model takes into account differences between studies and provides more conservative effects than fixed models (29). We applied *I^2^* statistics to detect heterogeneity across included studies (*I*^2^ < 50%, low heterogeneity; *I*^2^ = 50–75%, medium heterogeneity; and *I*^2^ > 75%, significant heterogeneity) (30). To recognize potential sources of heterogeneity, we carried out subgroup analyses and meta-regression analyses with the following factors: study design, sample size, study quality, and adjustment for potential confounding factors (total energy intake, education level, and physical activity). Sensitivity analyses were also performed to detect the impact of single research on the summary effect value by removing one study from the whole analysis in each turn (31). Ultimately, the risk of publication bias was examined by visual inspection through funnel plots, Egger’s linear regression test (32), and Begg’s rank correlation (33). We used Cohen’s Kappa statistics to evaluate reviewers’ consistency in data extraction and quality assessment (34). Stata version 11.0 software (StataCorp, College Station, TX) was applied for statistical analyses of our meta-analyses. A two-tailed *p* < 0.05 was considered significant.

## Results

### Search results

**Figure 1:**
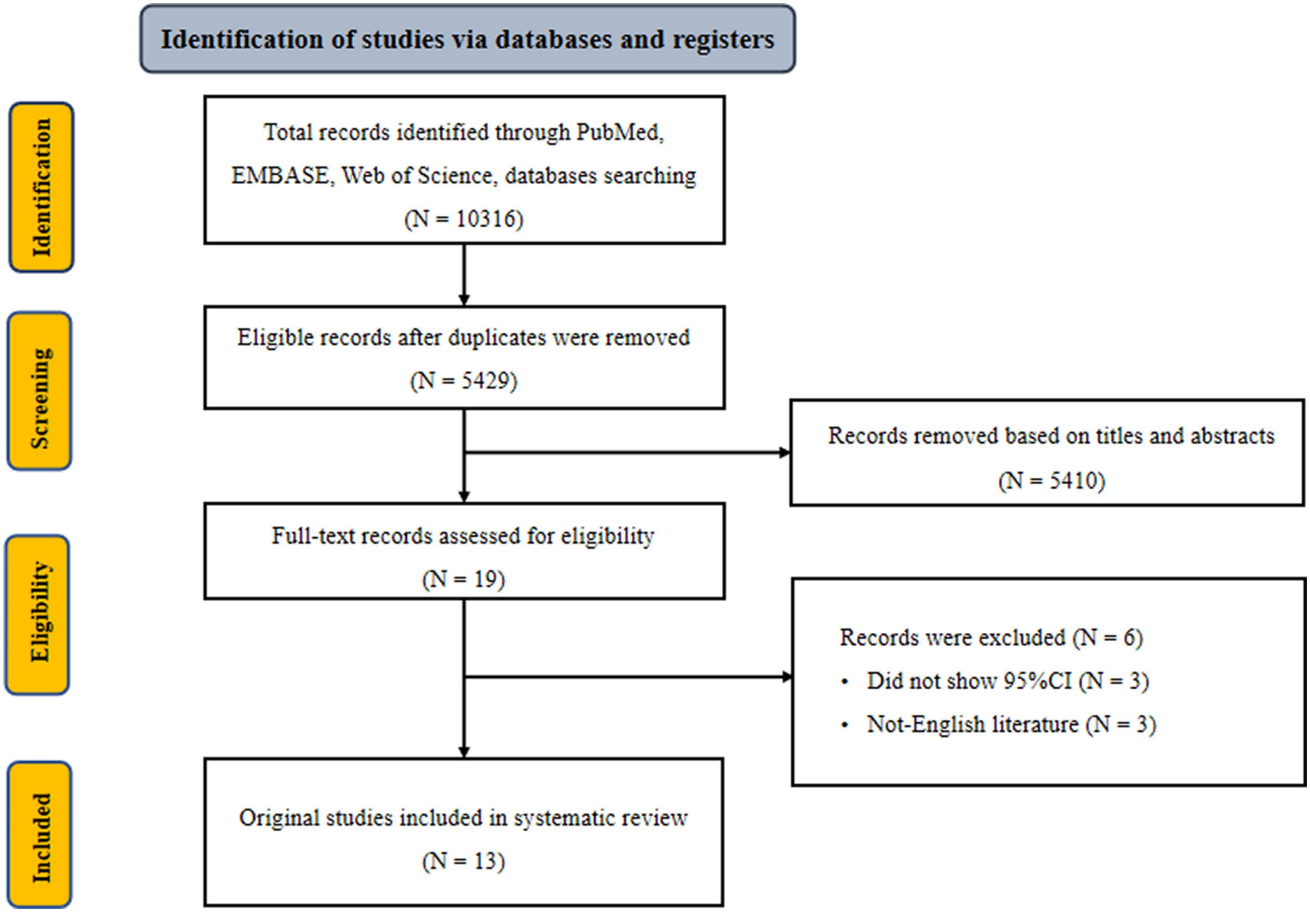
Flowchart of selection of studies for inclusion in meta-analysis on dietary pattern and hyperuricemia risk.

Initially, we retrieved 10,316 potentially eligible literature from three electronic databases, including PubMed, Embase, and Web of Science three electronic databases. After excluding duplicate literature, 5,429 records remained. Of these, 5,410 records were deemed ineligible after screening titles and abstracts. Nineteen full-text records were further reviewed. Six records were ruled out because of the following exclusion criteria: did not show 95% CI and non-English literature (35–40). The remaining 13 eligible records were included in our analysis (14–21, 41–45) (Figure 1). According to kappa coefficients, the authors’ agreement rate for data extraction, selection, and quality assessment was 20%.

### Study characteristics and quality assessment

The major characteristics of the original studies are shown in Table 1. All studies were conducted in China (14–21, 41–45). Two of these studies were cohort studies (17, 18), two were case–control studies (16, 41), and nine were cross-sectional studies (14, 15, 19–21, 42–45). All included studies were published from 2012 to 2023 and enrolled adults (18 years and over), with a total of 163,192 subjects. A validated food frequency questionnaire was used in the majority of studies (14–17, 19–21, 41–45), while the remaining studies used a 3-day 24-h food record (18). Table 2 lists the adjustment for confounders in the original studies. All included studies accounted for potentially important confounding factors like age, gender, etc. Several studies adjusted for additional confounding factors, such as education level (*n* = 9), total energy intake (*n* = 8), and physical activity (*n* = 7). Supplementary Tables S4–S6 summarized the results of the quality assessment of the included studies. Based on the NOS quality assessment, two cohort studies (17, 18) were rated as high quality, and two case–control studies (16, 41) were rated as medium quality. In line with the AHRQ quality evaluation, six studies were rated as good quality (16, 39, 41–44) and three studies were rated as fair quality (14, 15, 42–44).

**Table 1:** Characteristics of included studies.

First author, reference, year	Study design	Total number of subjects (age)	Dietary assessment	Methods defining dietary patterns	Dietary patterns and the main foods in the dietary pattern	Risk estimates (95%CI)
Luo et al. (2023) (19)	Cross-sectional study	12,318 (aged 18–79 y)	FFQ	FA with varimax rotation	*Healthy pattern*: vegetable, tubers, egg, legumes, and legume products *Traditional pattern*: pickled foods, meat, Chinese sauerkraut, and refined grain *Animal foods pattern*: animal organs, animal blood, preserved eggs, and processed meat products *Sweet foods pattern*: sugar-containing beverages, ice cream and candy, cake, fruit, or vegetable juice *Tea-alcohol pattern*: alcohol and alcoholic beverages, tea and tea beverages, and fish	OR: 0.68 (0.43, 1.08) OR: 0.49 (0.32, 0.74) OR: 1.42 (0.98, 2.08) OR: 1.69 (1.18, 2.42) OR: 1.24 (0.83, 1.90)
Wu et al. (2023) (21)	Cross-sectional study	84,653 (mean age: 51.7 y)	FFQ	PCA without varimax rotation	*Sichuan Basin pattern*: fish/seafood, poultry, eggs, dairy products, and fresh fruits *Yunnan-Guizhou Plateau pattern*: animal oil, rice, salt, preserved vegetables, and alcohol *Qinghai-Tibet Plateau pattern*: coarse grain, wheat products, tubers, and tea	OR: 1.05 (1.02–1.08) OR: 0.91 (0.88–0.94) OR: 1.02 (0.99–1.06)
Kong et al. (2023) (20)	Cross-sectional study	3,383 (age: 9-17y)	FFQ	PCA with varimax rotation	*Ultra-processed*: fried foods, drinks, snack foods, and processed meats *Plant-based*: nuts, dairy, fruits, whole grains, and pulses *Meat-based*: red meats, poultry, and refined grains Soup/seafood/egg: soup, fish and other seafood, and eggs *Vegetarian*: tubers, vegetables, soybeans, and soy products mushroom/animal organ: mushrooms, animal organs, and blood	OR: 0.99 (0.76, 1.31) OR: 0.92 (0.69, 1.22) OR: 1.21 (0.85, 1.71) OR: 1.02 (0.79, 1.32) OR: 0.79 (0.59, 1.07) OR: 1.13 (0.88, 1.45)
Li et al. (2022) (45)	Cross-sectional study	1,136 (age > 65 y)	FFQ	PCA with varimax rotation	*Animal-based and processed food*: refined rice and noodles, meat, and aquatic products *Traditional food*: whole grains, vegetables, and legume products 0vo-lacto vegetarian food: dairy products and fruits	OR: 1.24 (0.73–2.11) OR: 0.67 (0.47–0.97) OR: 0.56 (0.39–0.79)
Yang et al. (2022) (14)	Cross-sectional study	18,691 (mean age: 66.50 y)	FFQ	FA with varimax rotation	*Typical Chinese*: wheat, rice, and coarse grain *Modern Chinese*: bacteria, legume products, and mixed beans *Western*: western staples, snacks, and soft beverage Animal products and alcohol: organ, red meat, and alcohol Tuber and fermented vegetables: fermented vegetables and tuber	OR: 0.32 (0.28–0.37) OR: 0.81 (0.71–0.93) OR: 1.04 (0.93–1.17) OR: 1.49 (1.31–1.70) OR: 0.78 (0.69–0.88)
Zhou et al. (2022) (15)	Cross-sectional study	4,855 (age > 18 y)	FFQ	PCA without varimax rotation	*Plant-based*: fresh vegetables, fruits, dairy products, and legumes *Processed food diet*: snacks, desserts, and processed meats *Animal diet*: fish, shrimp, and meat	OR: 0.70 (0.56–0.87) OR: 1.19 (0.96–1.46) OR: 1.40 (1.13–1.74)
Li et al. (2022) (16)	Cross-sectional study	2,646 (mean age: 44.10 y)	FFQ	PCA with varimax rotation	*Meat-based*: Viscera, snacks and pastries, fish, shrimp, crab and shellfish, and fresh meat *Plant-based*: Mushrooms and algae, beans and their products, nuts, fruits, and vegetables *Local special*: marinated and smoked meat and grease	OR: 1.39 (1.04–1.90) OR: 1.14 (0.86–1.50) OR: 0.87 (0.66–1.15)
Zhang et al. (2021) (17)	Cohort study	20,766 (mean age: 39.91 y)	FFQ	PCA with varimax rotation	*Vegetables*: celery, cucumber, Chinese cabbage, green leafy vegetables, and pumpkin *Sweet food*: strawberry, kiwi fruit, persimmon, grape, pineapple, Western-style pastry, and cakes *Animal food*: animal organs, animal blood, animal liver, preserved eggs, and sausage	HR: 0.79 (0.72–0.87) HR: 1.22 (1.12–1.33) HR: 1.24 (1.13–1.37)
Shi et al. (2021) (18)	Cohort study	8,429 (mean age: 51.0 y)	3-day 24 h food record	PCA with varimax rotation	*Traditional southern*: rice, wheat, whole grain, pork, and fish *Modern dietary*: fruit, milk, eggs, and fast food	OR: 3.24 (2.61–4.01) OR: 1.14 (0.90–1.43)
Xia et al. (2018) (41)	Case–control study	1,422 cases 1,422 controls (mean age: 42.39y)	FFQ	FA with varimax rotation	*Sweet pattern*: Strawberry, kiwi fruit, persimmon, sweets, candied fruits, cookies, Chinese cakes, and salted eggs *Vegetable pattern*: cucumber, Chinese cabbage, celery, green vegetable, and eggplant *Animal foods pattern*: animal organ, animal blood, animal liver, preserved egg, and seafood	OR: 1.10 (0.89–1.37) OR: 0·88 (0.71–1.09) OR: 1.50 (1.20–1.87)
Liu et al. (2018) (42)	Cross-sectional study	1,893 (18–96 y)	FFQ	PCA with varimax rotation	*Plant-based*: Mushroom and algae food, vegetables, legumes, nuts, brawn, and bacon *Animal products*: Wheat and its products, fish, and fresh meat *Mixed food*: Snacks and dessert, animal giblets, other cereal, and tubers	PR: 1.03 (0.84–1.26) PR: 1.34 (1.06–1.70) PR: 0.97 (0.78–1.20)
He et al. (2017) (43)	Cross-sectional study	1,204 (45–59 y)	FFQ	PCA with varimax rotation	*Traditional Chinese*: pork, vegetables, and starchy tubers *Meat food*: beef/mutton, processed and cooked meat, cakes, and biscuits *Mixed food*: fresh fruits, fish and shrimps, and seafood	PR: 0.82 (0.43–0.92) PR: 1.48 (1.12–2.10) PR: 1.24 (0.93–1.84)
Zhang et al. (2012) (37)	Case–control study	187 Cases 187 Controls (20–59 y)	FFQ	PCA with varimax rotation	*Animal products and fried foods*: animal giblets, fried wheat products, and eggs *Western*: poultry, beverages, liquor, and alcohol *Soybean products and fruit*: starchy tubers, fruit, and soybean products	OR: 2.20 (1.19–4.08) OR: 1.18 (0.59–2.36) OR: 0.28 (0.15–0.53)

FA, factor analysis; FFQ, food frequency questionnaire; HR, hazard ratio; OR, odds ratio; PCA, principal component analysis; PR, prevalence ratio.

**Table 2:** Adjustment potential confounders of included studies.

First author reference, year	Adjustment for potential confounders in the primary analysis
Luo et al. (2023) (19)	Age, gender, education level, marital status, smoking status, drinking status, physical activity, overweight/obesity, hypertension, diabetes, and hyperlipidemia, and total energy.
Wu et al. (2023) (21)	Age, sex, area, ethnicity, marital status, education, income, occupation, smoking status, metabolic equivalent, energy, sweetened beverage, dietary supplements, spicy food, pepper food, insomnia symptoms, depressive symptoms, anxiety symptoms, menopause status for women, family history of cardiometabolic diseases, hypertension, stroke, hyperlipidemia, diabetes, and coronary heart disease
Kong et al. (2023) (20)	Sex, age, geographic region, family income, maternal education, family history of hyperuricemia or gout, physical activity, overweight or obesity, puberty status, total energy intake, and other dietary patterns.
Li et al. (2022) (45)	Age, living status, education level, smoking status, alcohol consumption, and total energy intake
Yang et al. (2022) (14)	Age, gender, BMI, urban and rural, income, education, marital status, smoking, alcohol drinking, static status, sleeping time, and total energy intake groups
Zhou et al. (2022) (15)	Gender, age, residence, education level, alcohol consumption, smoking status, BMI, hypertension, diabetes, and dyslipidemia
Li et al. (2022) (16)	Age, BMI, gender, and ethnicity
Zhang et al. (2021) (17)	Sex, age, BMI, smoking status, alcohol consumption status, education levels, employment status, household income, physical activity, family history of the disease (including CVD, hypertension, hyperlipidemia, and diabetes), depressive symptoms, MetS, hypertension, hyperlipidemia, diabetes, total energy intake, other dietary pattern scores, glomerular filtration rate, and high-sensitivity C-reactive protein
Shi et al. (2021) (18)	Age, gender, intake of energy, education, income, urbanization level, smoking, alcohol drinking, physical activity, overweight/obesity, hypertension, and diabetes
Xia et al. (2018) (41)	Sex, age, BMI, physical activity, energy intake, education level, household income, smoking status, drinking status, employment status, and metabolic syndrome status
Liu et al. (2018) (42)	Age group, gender, BMI, smoking, drinking, hypertension, and hyperlipidemia.
He et al. (2017) (43)	Gender, age, education level, physical activity level, smoking status, alcohol use, hypertension, BMI, and total energy intake
Zhang et al. (2012) (37)	Age, sex, education level, physical activity, smoking status, drinking status, BMI, and blood lipids

BMI, body mass index.

### Association between dietary pattern and hyperuricemia risk

In 11 studies (14–17, 19, 20, 41–45) assessing the association between animal-based food patterns and HUA risk, we observed that the highest adherence to animal-based food patterns was associated with the highest risk of HUA (Table 3). The overall OR was 1.36 (95%CI = 1.25–1.47) with low heterogeneity (*I*^2^ = 26.7%). Three studies (17, 19, 41) demonstrated that the highest adherence to sweet food patterns was significantly associated with an improved risk of HUA (OR = 1.24, 95%CI = 1.06–1.46, *I*^2^ = 0) (Table 3).

**Table 3:** Subgroup analyses for the association between dietary pattern and hyperuricemia risk.

	Plant-based dietary pattern					Animal foods dietary pattern				
	No. of study	OR (95%CI)	*I*^2^(%)	P*	P**	No. of study	OR (95%CI)	*I*^2^(%)	P*	P**
Overall	10	0.83 (0.72–0.94)	72.9	<0.01		11	1.36 (1.25–1.47)	26.7	0.19	
Subgroup analyses										
Study design					0.52					0.44
Cohort study	1	0.79 (0.72–0.87)	N/A	N/A		1	1.24 (1.13–1.37)	N/A	N/A	
Case–control study	2	0.52 (0.17–1.58)	91.2	<0.01		2	1.62 (1.20, 2.20)	23.9	0.25	
Cross-sectional study	7	0.87 (0.74–1.03)	70.3	<0.01		8	1.38 (1.27, 1.50)	1.5	0.42	
Sample size					0.55					0.59
≥ median	5	0.78 (0.73–0.84)	0	0.64		5	1.38 (1.25, 1.52)	35.8	0.18	
< median	5	0.84 (0.61–1.15)	81.8	<0.01		6	1.32 (1.12, 1.55)	30.2	0.21	
Study quality					0.46					0.07
High quality	6	0.76 (0.61–0.95)	75.2	<0.01		6	1.30 (1.13, 1.50)	41.9	0.13	
Moderate quality	4	0.88 (0.72–1.08)	76.1	<0.01		5	1.44 (1.31, 1.58)	0	0.94	
Dietary pattern identified					0.88					0.05
Factor analysis	3	0.80 (0.72–0.88)	0	0.50		3	1.49 (1.33–1.66)	0	0.97	
principal component analysis	7	0.82 (0.67–1.01)	80.8	<0.01		8	1.29 (1.18–1.42)	15.8	0.31	
Varimax rotation for dietary pattern					0.64					0.82
Yes	9	0.84 (0.73–0.97)	74.2	<0.01		10	1.35 (1.24, 1.48)	33.1	0.14	
No	1	0.70 (0.56–0.87)	N/A	N/A		1	1.40 (1.13–1.74)	N/A	N/A	
Adjustment for potential confounders										
Total energy intake					0.87					0.55
Yes	6	0.82 (0.74–0.92)	48.2	0.08		7	1.34 (1.20, 1.49)	41.4	0.10	
No	4	0.76 (0.52–1.12)	86.6	<0.01		4	1.41 (1.23, 1.62)	0	0.53	
Education level					0.13					0.99
Yes	8	0.77 (0.67–0.89)	78.7	<0.01		9	1.36 (1.23, 1.50)	41.1	0.09	
No	2	1.07 (0.91–1.26)	0	0.56		2	1.36 (1.13, 1.64)	0	0.85	
Physical activity					0.65					0.20
Yes	5	0.77 (0.60–0.99)	79.0	<0.01		6	1.33 (1.15, 1.53)	47.2	0.09	
No	5	0.85 (0.71–1.02)	71.4	<0.01		5	1.43 (1.30, 1.57)	0	0.91	

*P-Value for heterogeneity within each subgroup. **P-Value for heterogeneity between subgroups with meta-regression analysis.

**Figure 2:**
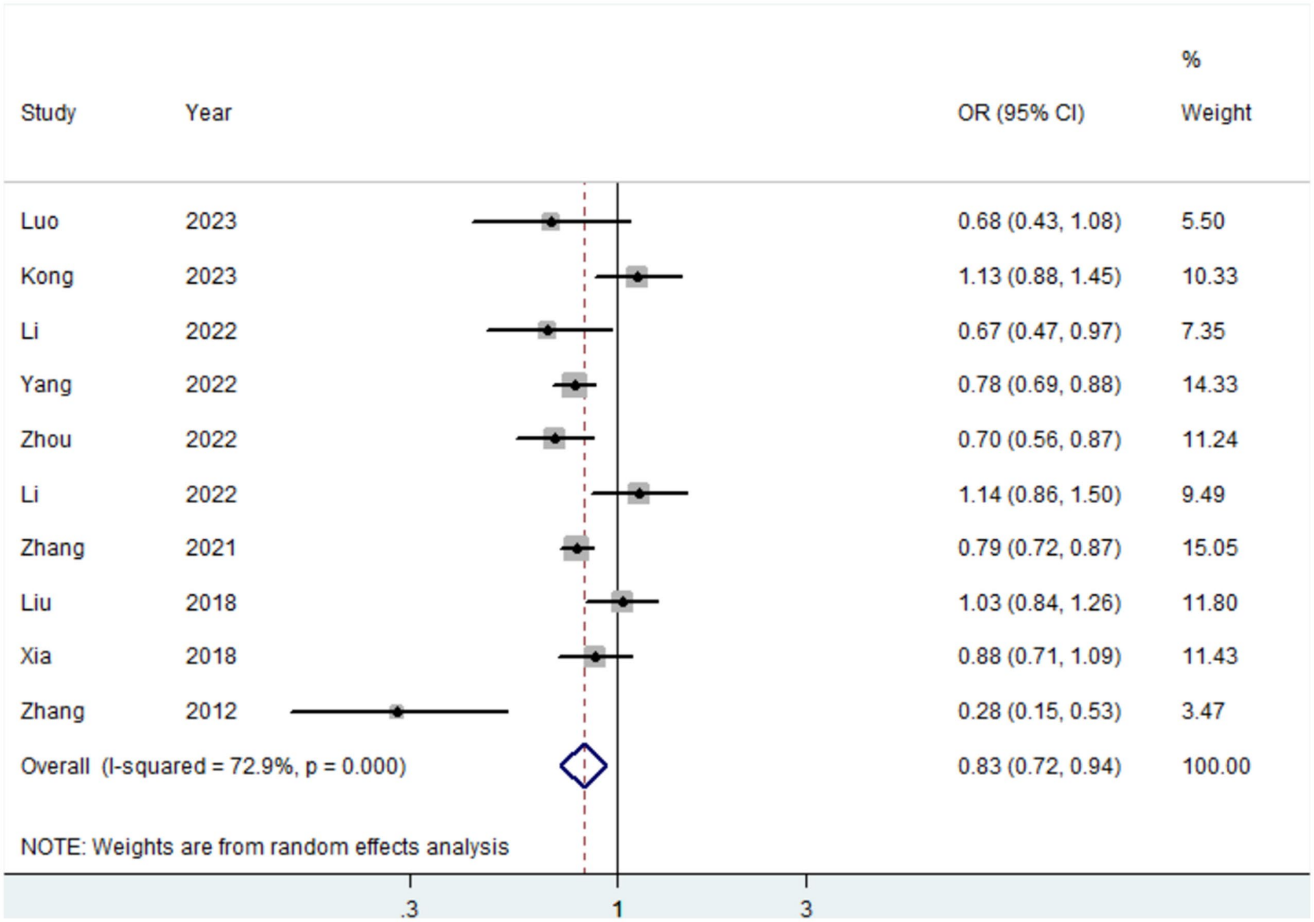
Forest plot (random-effects model) of dietary patterns with the risk of hyperuricemia.

**Figure 3:**
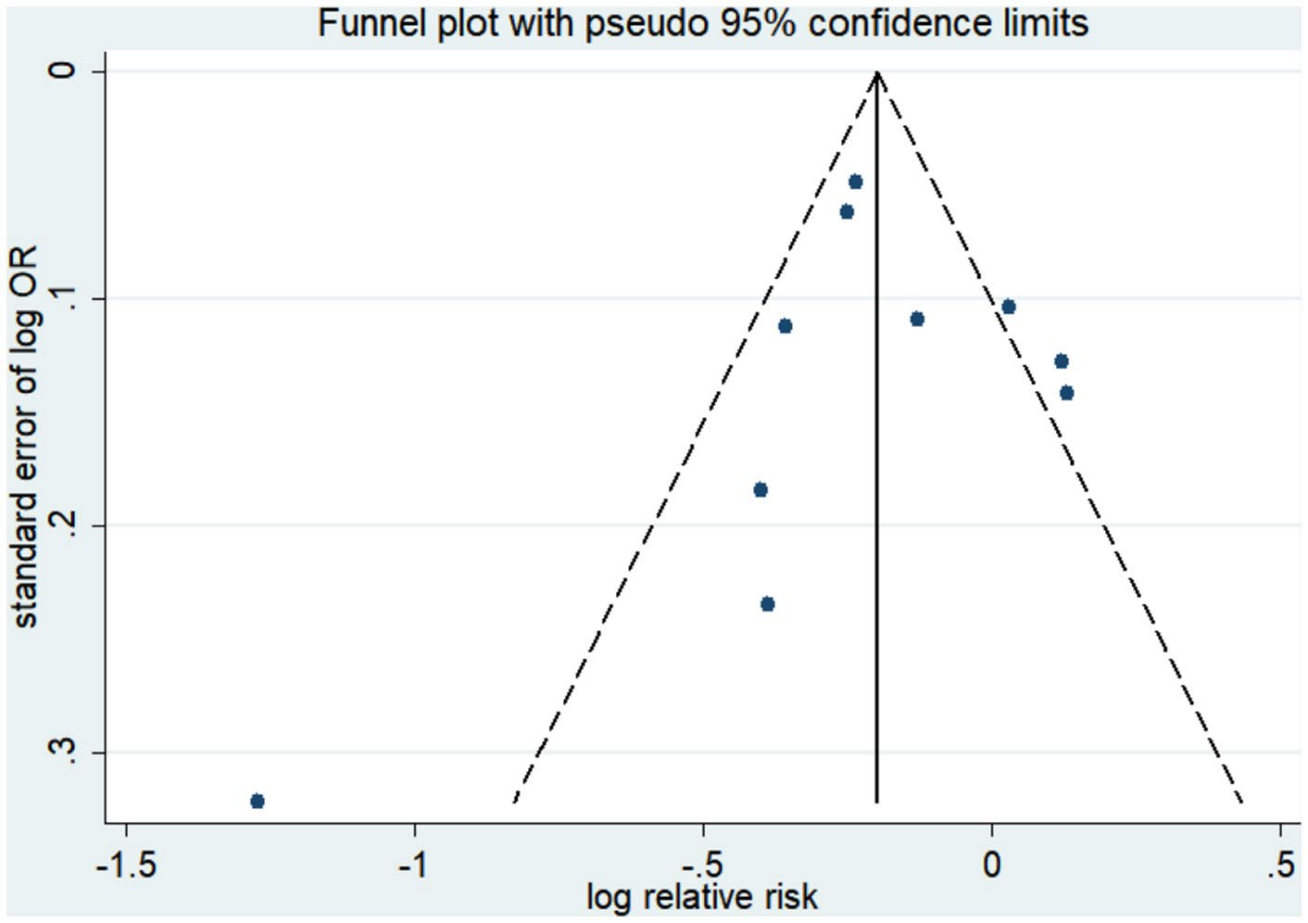
Funnel plot of publication biases of studies included in our meta-analysis focusing on the association between dietary patterns with the risk of hyperuricemia.

**Figure 4:**
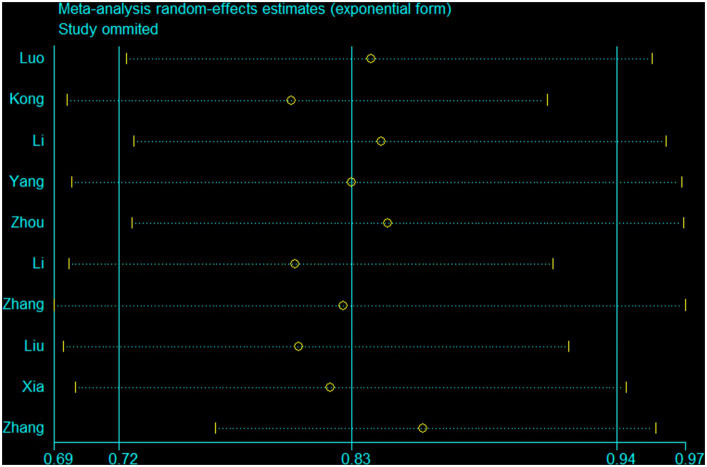
A sensitivity analysis was performed by removing each study in turn and recalculating the pooled odds ratio.

Ten studies (15–17, 19, 20, 41, 42, 44, 45) were conducted to assess the association between plant-based patterns and HUA risk, and we observed that high adherence to plant-based dietary patterns was associated with a lower risk of HUA (OR = 0.83, 95% CI = 0.72–0.94, and *I*^2^ = 72.9%) (Figure 2). The comprehensive findings of subgroup analyses and meta-regression analyses of the association between plant-based patterns and HUA risk are shown in Table 3. Among the three subgroups of study design, cohort studies (OR = 0.79, 95% CI = 0.72–0.87) demonstrated an inverse association between adherence to plant-based dietary patterns and HUA risk, whereas case–control studies (OR = 0.52, 95% CI = 0.17–1.58) and cross-sectional studies (OR = 0.87, 95% CI = 0.74–1.03) indicated no statistical association. Subgroup analysis according to sample size and study quality revealed that higher adherence to plant-based patterns compared with lower adherence was associated with a lower risk of HUA in sample size ≥ median (OR = 0.78, 95%CI = 0.73–0.84) and high quality (OR = 0.76, 95%CI = 0.61–0.95) subgroup. Among subgroup analyses based on adjustment for potential confounders, we observed inverse correlations between higher adherence to plant-based dietary patterns and HUA risk in adjustment for total energy intake (OR = 0.82, 95%CI = 0.74–0.92), education level (OR = 0.77, 95%CI = 0.67–0.89), and physical activity (OR = 0.77, 95%CI = 0.60–0.99). Besides, there is no evidence of heterogeneity between the aforementioned subgroup analyses in the results of the meta-regression analysis. Furthermore, no publication bias was detected (Egger’s *p* = 0.83 and Begg’s *p* = 0.36; Figure 3). Sensitivity analysis showed no change in pooled estimates of the effect of plant-based patterns on the risk of HUA after successive exclusion of each article (Figure 4).

## Discussion

Our meta-analysis with 163,192 participants was the most comprehensive topic to systematically evaluate the association between *posteriori* dietary patterns and HUA risk. Following the data analysis, the current evidence indicated that adherence to the plant-based pattern was associated with a reduced risk of HUA, while adherence to the animal-based food pattern and sweet food pattern was the opposite. No significant association was detected between modern dietary patterns and HUA risk.

The inverse association between the plant-based pattern and HUA risk was well established in subgroup analysis stratified by cohort study, sample size ≥ median, and dietary pattern identified in factor analysis. Some previous studies were consistent with the aforementioned findings (15, 17, 44). Furthermore, a meta-analysis of randomized controlled trials also indicated that DASH diet interventions similar to a plant-based pattern significantly reduced serum uric acid (46). However, several studies were disparate with the above inverse association (16, 41, 42). For example, a cross-sectional study including 2,646 subjects conducted by Li et al. found that plant-based pattern was not significantly associated with HUA risk (16). Another cross-sectional study performed by Liu et al. with 1,893 (42) and a case–control study including 187 cases and 187 controls performed by Xia et al. (41) had the same findings. Besides, two cross-sectional studies with large sample sizes also found that plant-based dietary patterns were not associated with the risk of HUA (19, 20). The difference in the main finding might be explained by the small sample size and poor quality of cross-sectional and case–control studies compared to cohort studies, which might be the source of heterogeneity of association between the plant-based pattern and HUA risk. Besides, in the subgroup analysis of dietary pattern identified methods, compared with factor analysis to determine dietary patterns, principal component analysis identified dietary patterns as a possible source of heterogeneity. Therefore, the aforementioned notions require stronger evidence by prospective studies with a large sample in the future.

There are a number of possible explanations or underlying biological mechanisms for the beneficial effect of the plant-based pattern in preventing HUA. Similar to the healthy Mediterranean pattern, the plant-food pattern is characterized by a high intake of fresh fruits, vegetables, legumes, and whole grains. Notably, high levels of vitamins like vitamin C, folate acid, and minerals in fruits and vegetables may be effective in preventing HUA (47–49). For example, since vitamin C and uric acid are reabsorbed via anion exchange transport in proximal tubules, the increase of vitamin C may compete with the reabsorption of uric acid (47, 48); folic acid may decrease uric acid production by deactivating the enzymes that oxidize hypoxanthine into xanthine (49). In addition, dietary fiber in fruits and vegetables and isoflavones in soy products had important benefits for a lower risk of HUA (50, 51). Dietary fiber promotes uric acid excretion by binding to uric acid in the gut (50). Soybean may decrease uric acid production by deactivating xanthine oxidoreductase, a key enzyme in the oxidation of hypoxanthine to xanthine (51). Magnesium is an essential mineral for the human body. A study conducted in 2020 found that plasma magnesium levels were inversely associated with the risk of HUA and increased uric acid concentrations (52). Therefore, we suppose that magnesium mainly reduces uric acid concentration by increasing the excretion of uric acid; however, the specific mechanism remains to be explored (52, 53). Copper is another essential trace mineral for humans. Copper and molybdenum have antagonistic effects. Therefore, the mechanism by which copper affects uric acid concentration is that copper inhibits the activity of xanthine oxidase and dehydrogenase, hindering the oxidation of purine to uric acid, and thereby reducing the concentration of serum uric acid (54).

Animal dietary patterns containing large amounts of animal organs, animal blood, and animal liver are associated with an increased risk of HUA. Overall results and subgroup analysis all supported the aforementioned findings. This association comes down to the following. First, animal-based food patterns are high in purines and the accumulation of purines leads to higher uric acid levels (41, 55). This potential mechanism was demonstrated in metabolic experiments in animals and humans that examined the effect of artificially short-term loading purified purines on serum uric acid levels (56). Second, a high intake of animal-based food patterns means a higher intake of energy, which in turn leads to obesity. It was found that in the general population, obesity or centripetal obesity were significantly positively correlated with HUA risk (57, 58). A study performed on participants with visceral fatty obesity showed that elevated uric acid levels were strongly influenced by their overproduction and reduced excretion and clearance of uric acid in the urine (59). In addition, visceral fat accumulation induces plasma-free fatty acids to flow into the living body and the hepatic portal vein, thereby stimulating triglyceride synthesis and subsequently leading to a related surge in uric acid production by activating the uric acid synthesis pathway (60, 61). Third, animal-based food patterns are high in pro-inflammatory nutrients. A previous study indicated that a high pro-inflammatory diet score was associated with an increased risk of HUA (62). HUA can be further caused by high uric acid production or abnormal excretion of uric acid under inflammatory conditions (62). Since the physiological mechanism of hyperuricemia caused by inflammation remains unclear, future studies should aim to address this topic.

The overall result demonstrated that greater adherence to sweet food patterns was significantly positively linked with an increased risk of HUA. A prospective cohort study including 20,766 subjects also revealed a positive association between greater adherence to the sweet food pattern and an increased risk of HUA (OR = 1.69, 95% CI: 1.18–2.42) (17). Moreover, Luo et al. conducted a cross-sectional study involving 12,318 subjects and observed that subjects with greater adherence to sweet food patterns had an improved risk of HUA (41). However, a case–control study (including 1,422 controls and 1,422 cases) conducted by Xia et al. indicated that there is no significant association between sweet food dietary patterns and HUA (37). We consider that the inconsistencies are mainly due to differences in overall variance. Xia et al. explained 22.6% variance, which is slightly lower than Luo et al. and Zhang et al., which, respectively, explained 38.93 and 29.2% of the overall variance. The potential mechanism by how sweet food patterns are associated with an improved risk of HUA may be brought down to fructose in sweet foods. Fructose metabolism converts adenosine triphosphate to inosine monophosphate, which activates the catabolic pathway that leads to the production of uric acid (63, 64). Experimental studies in humans and animals have indicated a short-term increase in uric acid concentrations following fructose intake or infusion (63, 65). Besides, studies have suggested that excessive fructose intake can alter the composition of the gut microbiota, which in turn affects UA metabolism (66).

Several strengths are worth to be highlighted for our meta-analysis. First, as far as we know, our topic is the most comprehensive one to systematically summarize and analyze the associations between a variety of *posteriori* dietary patterns and HUA risk. Moreover, we conducted a diverse array of subgroup analyses, like dietary pattern identified methods, varimax rotation for dietary pattern, and confounding factors adjustment to further explore the sources of heterogeneity. Second, a rigorous literature search, study selection, and data extraction were performed by two independent authors and the original articles included in this meta-analysis are all medium-high quality literature, which makes the results more reliable. Third, sensitivity analysis and meta-regression analyses were conducted according to the characteristics of the study and the adjustment of major confounding variables to detect the robustness of the results.

Nevertheless, some caveats deserve to be outlined for our analyses. First, our meta-analysis included a total of five cross-sectional studies, and due to the nature of cross-sectional studies, the causal relationship between dietary patterns and HUA is limited. Second, dietary assessment is done using the food frequency questionnaire or 24-h food record, so there is an inevitable recall bias. Third, in the original studies, subgroup analyses on gender, age, region, and HUA type were rarely performed, which resulted in limited secondary analysis results. Fourth, despite the protective effect observed in the plant-based pattern, there was considerable heterogeneity, and further studies need to explore the source of heterogeneity. Fifth is the inherent nature of observational studies. Although the included studies have adjusted for a large number of confounding factors, other residual confounding factors are inevitable. Lastly, only published literature was included and analyzed, while other unpublished and gray literature content that meets our criteria might be neglected.

## Conclusion

Our meta-analysis reveals that adherence to the plant-based pattern is a beneficial factor for HUA risk, while adherence to the animal-based food pattern and sweet pattern is an unfavorable factor. Future high-quality studies with larger sample sizes and longer follow-up periods are needed to further validate our findings.

## Author contributions

Z-YW, Y-FW, and W-PJ collection of data. Z-YW, Y-FW, Y-HS, and W-PJ wrote the first draft of the manuscript and edited the manuscript. All authors contributed to the article and approved the submitted version.
